# Notes and News

**Published:** 1897-01-30

**Authors:** 


### NOTES AND NEWS.

(COLLECTED AND COMMUNICATED.)

THE miseries in Bombay continue to increase. The supply of gas is threatened owing to the panic amongst the operatives. The want of medical attendance and nursing is reported as very great, and it is suggested that an additional staff of medical men and nurses is needed as much as anything.

AT St. Andrews University the Marquis of Bute has provided funds for the erection of new medical classrooms, and has endowed a lectureship in physiology for two years, to which Miss Umpherston has been appointed. Miss Umpherston is a L.R.C P. and L.R.C.S. Edinburgh, and L.F.P.S. Glasgow, and was at one time assistant house surgeon at the New Hospital for Women.

THE committee of the Middlesborough New Asylum are advertising for a medical superintendent, and candidates are asked to state salary required. This would lead to the inference that applicants have to tender for the appointment, and tendering for an appointment is opposed to correct medical ethics. It would be strange if medical men apply for the post until the form of advertisement is altered.

WITH a view to establishing some standard of fees which can be referred to in cases where medical men are sued for overcharge, an official tariff has been instituted in Prussia. The minimum and maximum charges are tabulated, and embrace most of the exigencies of practice. The fees for serious operations would meet with little favour amongst English surgeons, the maximum charge being fixed as low as £25.

A BRANCH of the Bristol Dispensary has been opened at Bedminster, a populous and growing district. The building contains a large general waiting-room, adjoining the dispensary, with doctors' consulting-rooms and smaller waiting-room on the other side. The first floor is arranged as a residence for the dispenser. The cost, including site, is estimated at about £2,400. Part of this sum has been provided from the investments held by the parent institution, whilst the public are appealed to for the remaining expenditure required.

THE Royal Commission on Tuberculosis has held six sittings during the last fortnight, when a large number of witnesses were examined, representing the meat and cattle trade, and numerous inspectors and medical officers of health.

THE Cardiff Infirmary has secured £3,300 as the results of the recent bazaar held on its behalf, and to this sum the Marquis of Bute has added a donation of £1,000. The committee have adopted a novel course of encouraging collections for the Diamond Jubilee commemoration by offering prizes of jewellery to the ladies making the largest collection to endow a cot.

THE arrangements which are being made for the Montreal meeting of the British Medical Association, which begins on August 31st next, seem very complete. A Finance Committee is actively engaged in arranging the financial part of the business, and it is expected that ample funds will be received. The sum of 2,000 dols. has been received from the Dominion Government, 2,000 dols. from the Provincial Government, and 3,000 dols. from the city of Montreal. The Museum Committee have secured the Victoria Rink for the exhibition. In the various hotels there is accommodation for considerably more than a thousand guests, at prices ranging from two to five dollars per day, including board and lodging, three to four dollars being the more common price. First-class board and lodging can be had in the immediate vicinity of McGill University at from ten to twelve dollars a week, and quite comfortable accommodation in other parts at considerably lower rates. As for travel, such arrangements have been made that it will be possible to travel from Liverpool to Montreal, reside in Montreal during the time of the meeting, and return to Liverpool at the expense of, say, £25, including not only board and lodging on the steamer and in Montreal, but also excursions in its immediate vicinity and to Niagara. Verily a pleasant outing!

A VERY important step has been taken by the governors of St. Mary's Hospital with the object of mitigating the ever-growing difficultyof the out-patients. They have decided to appoint a new medical officer, who shall see all new cases and select from them twenty-five each day to be admitted to the consulting-room of the out-patient physician. He will also treat any case of immediate urgency, and order medicine, for that occasion only, to all who come provided with letters, even although they be not thought fit for admission as out-patients. The officer to be appointed will be of physician's rank, the membership of the Royal College of Physicians being obligatory. He will be appointed for one year, and be eligible for re-election for one year only, and will receive remuneration at the rate of £75 per annum. It is hoped that by this appointment it will be possible to put an effectual check on the excessive number of patients who have hitherto crowded the rooms of the out-patient physicians, and at the same time, by placing the selection of the cases in the hands of a responsible official, to remove the liability to error, and to consequent reproach, which must always exist where the selection is made by unqualified men or officers in subordinate positions. It certainly seems to be a move in the right direction.

